# Measuring Human Pavlovian Reward Conditioning and Memory Retention After Consolidation

**DOI:** 10.1111/psyp.70058

**Published:** 2025-04-25

**Authors:** Yanfang Xia, Huaiyu Liu, Oliver K. Kälin, Samuel Gerster, Dominik R. Bach

**Affiliations:** ^1^ Computational Psychiatry Research, Department of Psychiatry, Psychotherapy, and Psychosomatics Psychiatric University Hospital Zurich, University of Zurich Zurich Switzerland; ^2^ University of Bonn, Transdisciplinary Research Area Life and Health, Centre for Artificial Intelligence and Neuroscience, University of Bonn Bonn Germany; ^3^ Donders Institute for Brain, Cognition and Behaviour Radboud University Nijmegen Nijmegen the Netherlands; ^4^ Department of Psychiatry Radboud University Medical Centre Nijmegen the Netherlands; ^5^ Department of Imaging Neuroscience, UCL Queen Square Institute of Neurology University College London London UK

**Keywords:** associative learning, Pavlovian reward conditioning, psychophysiological measure, reward

## Abstract

While a body of literature has addressed the quantification of aversive Pavlovian conditioning in humans, Pavlovian reward conditioning with primary reinforcers and its recall after overnight consolidation remain understudied. In particular, few studies have directly compared different conditioned response types and their retrodictive validity. Here, we sought to fill this gap by investigating heart period responses (HPR), skin conductance responses (SCR), pupil size responses (PSR), and respiration amplitude responses (RAR). We conducted two independent experiments (*N*_1_ = 37, *N*_2_ = 34) with a learning phase and a recall phase 7 days later. A visual conditioned stimulus (CS+) predicted fruit juice reward (unconditioned stimulus, US), while a second CS− predicted US absence. In experiment 1, model‐based analysis of HPR distinguished CS+/CS−, both during learning (Hedge's *g* = 0.56) and recall (*g* = 0.40). Furthermore, model‐based analysis of PSR distinguished CS+/CS− in early trials during recall (*g* = 0.69). As an out‐of‐sample generalization test, experiment 2 confirmed the result for HPR during learning (*g* = 0.78) and recall (*g* = 0.55), as well as for PSR during recall (*g* = 0.41). In contrast, peak‐scoring analysis of PSR yielded low retrodictive validity. We conclude that in our Pavlovian reward conditioning paradigm, HPR is a valid measure of reward learning, while both HPR and PSR validly index the retention of reward memory.

## Impact statement

Pavlovian appetitive conditioning is an important experimental model, but its quantification remains heterogeneous. In a study with visual CS and primary US (fruit juice), we show that heart period response (HPR) robustly and replicably measures reward learning, and the retention of reward memory after 7 days. These findings may be beneficial for future work on reward learning and its memory retention.

## 1 Introduction

Learning to predict rewarding events plays an important role for many biological organisms. A quintessential paradigm to study this in the laboratory is Pavlovian reward conditioning (Martin‐Soelch et al. 2007; Pavlov 1927). Here, an initially neutral stimulus (conditioned stimulus, CS) is repeatedly paired with a naturally appetitive event (unconditioned stimulus, US). After some trials, CS comes to elicit some behavioral or physiological response (conditioned response, CR). In turn, this CR is then taken to index learning of the association, and when it is elicited after a delay, it is taken as indicative of reward memory. In human conditioning research, encompassing both reward and aversive conditioning (Lonsdorf et al. 2017), learning is usually indexed by the difference between responses elicited by CS+ and those elicited by a CS−, which predicts the absence of the US, in a paradigm known as differential learning.

Several psychophysiological CRs have been suggested to reflect human reward learning. Specifically, quantification of human reward conditioning has been based on SCR (Exner et al. 2021; Klucken et al. 2019), cardiac responses (Ebrahimi et al. 2019, 2019; Hermann et al. 2000; Pietrock et al. 2019; Sayão et al. 2021; Wardle et al. 2018), startle eyeblink (Andreatta and Pauli 2015; Hermann et al. 2000; Stussi et al. 2018), postauricular reflex (Ebrahimi et al. 2019; Pietrock et al. 2019; Stussi et al. 2018), and pupil dilation (Bray et al. 2008; Pietrock et al. 2019; Pool et al. 2019; Prévost et al. 2013; Reinhard and Lachnit 2002; Schad et al. 2020; Seymour et al. 2007). Notably, evidence for CS+/CS− differences in these observables is inconsistent between studies. However, experimental protocols are also heterogenous and differ by the type of paradigm (delay or trace conditioning), the type of CS and US, including primary and secondary reinforcers, CS–US intervals, and reinforcement schedules (Exner et al. 2021; O'Doherty et al. 2003; Wardle et al. 2018). Consequently, the interpretation of diverging results is difficult. Some previous work has directly compared different CRs, again with heterogeneous results. One study reported similarly small effect sizes in corrugator EMG, zygomaticus EMG, and SCR (Wardle et al. 2018); another one showed a large effect in HPR and smaller effects in pupil dilation, gaze patterns, with no effect in SCR or startle eyeblink (Pietrock et al. 2019); and a third one a larger effect in SCR than in startle eyeblink (Andreatta and Pauli 2019). Furthermore, it is unclear whether any of these results would generalize to the assessment of memory retention after overnight consolidation. This question is relevant in the context of pharmacological and noninvasive intervention studies (Ojala et al. 2022; Wehrli et al. 2023, 2024; Xia et al. 2024). It also holds significant importance for preclinical studies in the context of experimental psychopathology. Here, reward learning is often taken as a model of addiction symptoms, such as cue‐induced drug craving (Keiflin and Janak 2015).

Thus, the present work aimed to identify the most sensitive psychophysiological CR to quantify reward learning and retention after overnight consolidation. We employed a Pavlovian reward conditioning paradigm with a primary reinforcer, in which participants learned CS–US contingencies in a learning session, and retention was tested after seven days in a recall test. Our outcome variables were based on four observables: SCR, PSR, HPR, and RAR.

To determine the most sensitive CR, we used a calibration approach. Put simply, this assumes a priori that participants do acquire a CS–US association and evaluates different putative CR by their ability to reproduce this, as indexed by retrodictive validity (Bach et al. 2020; Bach 2023). In our case, retrodictive validity can be expressed as the effect size to distinguish CS+/CS−. To protect ourselves against overfitting to peculiarities of small samples, we employed an exploration‐confirmation approach as in previous work on these observables (Castegnetti et al. 2016, 2017; Korn et al. 2017; Xia et al. 2022). Specifically, in experiment 1 we explored different CR indices derived from all observables in their sensitivity to distinguish CS+/CS−. We retained all indices that yielded an effect size of Hedge's *g* > 0.5 and confirmed them in experiment 2. This effect size was chosen a priori to be large enough to be usable in intervention studies, where the maximum achievable effect size is constrained by the effect size in the control group. In addition, we heuristically report smaller effect sizes in conjunction with significant *p*‐values (without correction for multiple comparisons).

## 2 Method

### 2.1 Sample Size and Participants

Power analysis for experiments 1 and 2 was performed using G*Power 3.1.9.7 (Faul et al. 2007). We analyzed a one‐sided paired *t*‐test, which is appropriate since we had directional hypotheses. As a result, 34 participants were needed to achieve our a priori chosen effect size of Cohen's *d* > 0.5 (which approximates Hedge's *g*) with 80% power and an alpha level of 0.05. We recruited 37 participants in experiment 1 to allow for drop‐outs due to early termination and data exclusion. For experiment 2, power analysis showed that 32 participants were needed based on the HPR effect size reported in experiment 1 with 80% power. We recruited 34 participants in experiment 2 to allow for drop‐out.

Healthy participants were recruited from the student and general population in Zurich. The governmental ethics committee approved the study (KEK‐ZH‐2013‐0118). All participants provided informed consensus using a form approved by the ethics committee and received monetary compensation based on experiment duration. See Table 1 for details of demographics and general information.

**TABLE 1:** Demographics and general information for experiments 1 and 2.

	Phase	Experiment 1	Experiment 2
Participants completed per protocol	Learning	37 (26 women)	34 (20 women)
	Recall	37 (26 women)	33 (19 women)
Participants excluded per protocol	Learning	0	0
	Recall	0	1^a^
Age (full sample)	Learning	24.19 (4.20)	24.91 (4.11)
	Recall	24.19 (4.20)	24.94 (4.17)
Drink fasting time (hours)	Learning	7.70 (3.69)	7.46 (3.73)
	Recall	5.18 (3.05)	6.52 (3.71)
Food fasting time (hours)	Learning	11.05 (3.89)	10.84 (3.12)
	Recall	11.23 (5.43)	11.79 (3.25)
Hunger (%)	Learning	67.64 (26.06)	67.86 (20.00)
	Recall	70.38 (28.25)	78.99 (20.56)
Thirst (%)	Learning	69.85 (20.23)	67.94 (19.75)
	Recall	70.92 (23.75)	75.92 (15.92)
Favorite juice rating (%)	Learning	89.42 (13.38)	93.08 (8.89)
Selection of apple juice	Learning	8	4
Selection of mango juice	Learning	10	13
Selection of multivitamin juice	Learning	14	10
Selection of orange juice	Learning	5	7
	Recall		
Trait anxiety (STAI‐T)		36.59 (8.03)	38.82 (10.43)
State anxiety (STAI‐S) before learning		33.05 (6.98)	34.94 (8.89)
State anxiety (STAI‐S) before recall test		31.84 (7.05)	34.00 (7.53)

*Note:* Table shows mean (standard deviation), except in rows “Participants completed per protocol” and “Participants excluded per protocol”.

^a^ One participant did not return to the recall test due to illness.

### 2.2 Experimental Procedure

#### 2.2.1 Overview

**FIGURE 1:**
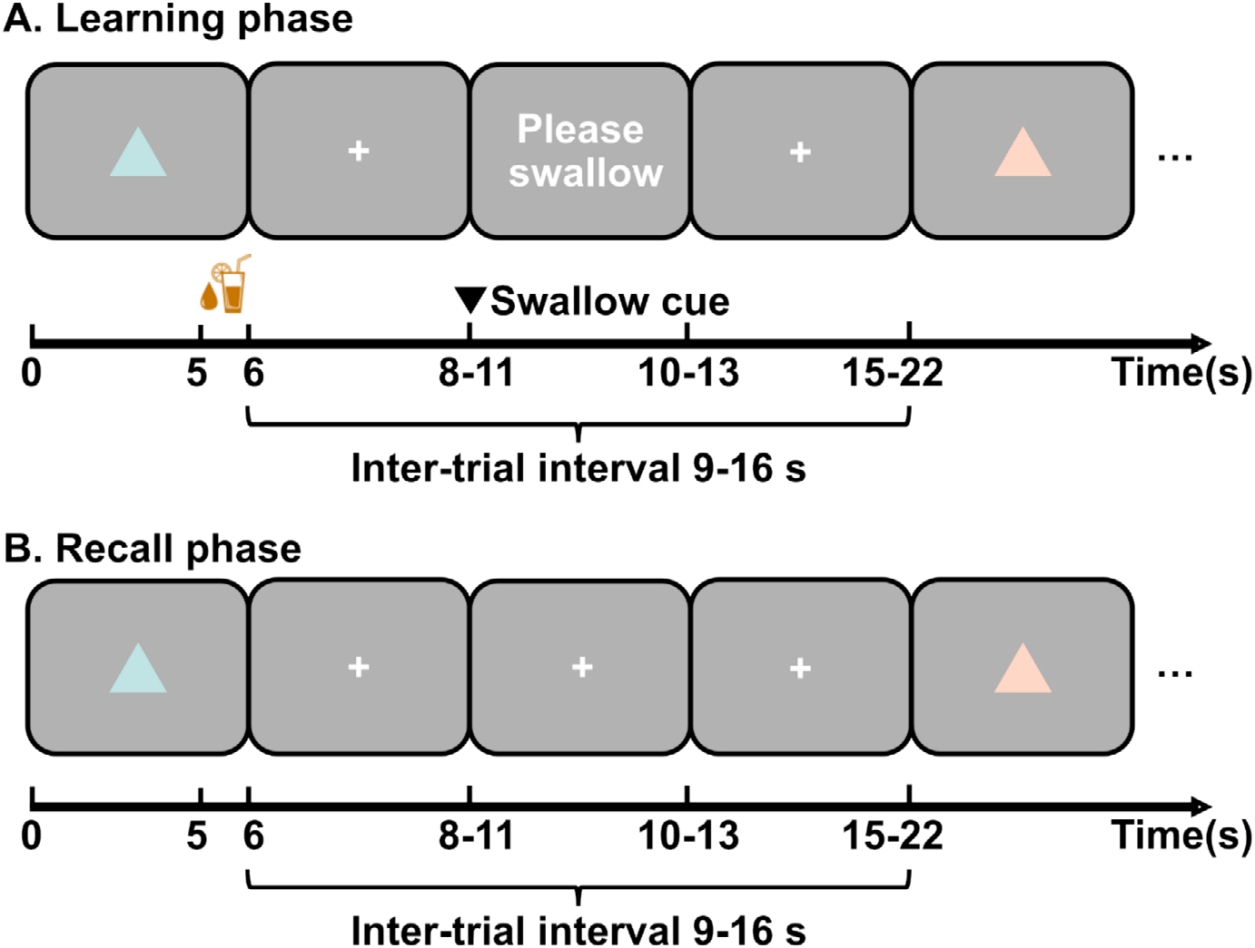
(A) A sample trial of the reward learning phase, in which participants learned to associate a colored triangle (CS+) with a fruit juice reward (US). The reinforcement rate was 50% and another colored triangle was never reinforced (CS−). In each trial, participants had to press the right or left arrow key to indicate the presented CS. Participants were informed about wrong or no key presses during the first 2 s of the intertrial interval (ITI). The signal to swallow was presented at a random time between 2 and 5 s after CS offset for 2 s. The duration of the ITI was randomly sampled between 9 and 16 s. (B) A sample trial of the recall phase, which is identical to the learning phase except that there was no US.

Both experiments followed the same procedure. Before arrival, to enhance US craving, participants were asked to fast from food for at least 6 h and from drinks for at least 4 h before arrival. Upon arrival, participants provided informed consent and were given instructions about the entire experiment. Next, participants completed the State–Trait‐Anxiety‐Inventory (STAI) and watched a 4‐min priming video with presentation of delicious food and drink images. After the video, they reported their food‐ and drink‐fasting duration (Table 1) and rated their hunger and thirst levels on a visual analogue scale (VAS) with endpoints 0–100. They chose their favorite flavor among apple, orange, mango, and multivitamin juices, and then rated all four juices on a VAS with endpoints 0–100, which was consistent with the categorical selection for all participants. The chosen favorite juice was then used as US in the subsequent Pavlovian reward conditioning task (Figure 1). This conditioning task was conducted in the morning between 8:00 a.m. to 12:00 p.m. to ensure that synaptic consolidation was largely completed before sleep for all participants. After conditioning, participants completed a computer‐based questionnaire about awareness of CS–US contingency (“How likely were you to receive a sip of juice when looking at this triangle today”, “How likely were you to receive a sip of juice when looking at this triangle last week?”) as well as experienced arousal and valence to all CS. Seven days later, participants came back for a recall test after the same fasting procedure. For logistical reasons, this recall test was conducted in the afternoon between 1:00 pm to 5:00 pm. They completed the state anxiety part of the STAI and watched the same priming video as described earlier, followed by fasting time reporting and ratings for hunger and thirst. Subsequently, they completed the recall task with a follow‐up computer‐based questionnaire about CS–US contingency (‘How likely were you to receive a sip of juice when looking at this triangle last week?’) and ratings on arousal and valence to all CS conditions.

#### 2.2.2 Pavlovian Reward Conditioning Task

All experiments were presented via MATLAB R2021a (The Math Works; https://www.mathworks.com/products/matlab.html) using the Cogent 2000 toolbox (http://www.vislab.ucl.ac.uk/Cogent). Each phase of the task included two blocks, with 24 CS+ and 24 CS− trials per block, resulting in 96 trials per phase in total. Trial order was randomized with the constraint that there were no more than three trials with the same CS, or more than three US, in a row. In the learning phase, the CS+ was reinforced 50% of the time, whereas the CS− was never reinforced. The first CS+ trial in each block was always reinforced. In the recall phase, both CS conditions were never reinforced. The instruction for the learning phase was “In this experiment, you will see differently colored triangles and receive a sip of juice now and then. You will notice that depending on the triangle you will receive a sip of juice more or less frequently”; the instruction for the recall phase was “Today the same triangles will be presented again”. In both phases, no explicit CS–US contingency instructions were given to participants.

Each trial started with a 6‐s CS presentation, followed by an intertrial interval (ITI) during which a fixation cross was presented. ITI duration was a random integer between 9 and 16 s. In reinforced CS+ trials, a sip of fruit juice was automatically delivered into participants' mouth 5 s after CS+ onset. To avoid artifacts in the psychophysiological recordings, participants were tasked to keep the juice in their mouth and swallow during the ITI when signaled. This signal appeared at random between 2 and 5 s after CS offset for 2 s. In order to keep the participants attentive during the task, they were asked to press a specific key associated with each CS. If participants did not respond or pressed the incorrect key, error feedback was given during the first 2 s of the ITI.

#### 2.2.3 Stimuli and Apparatus

The 4‐min priming video consisted of 63 appetitive food and drink images; each image was displayed for 4 s. All images were selected from an online image repository (https://unsplash.com/t/food‐drink) with a CC0 license.

Red and blue isoluminant triangles (RGB: [0.753, 0.894, 0.894], [1, 0.843, 0.776]) were randomly allocated to CS+ and CS− across participants. Both CS were presented for 6 s at the center of an isoluminant gray computer screen (RGB: [175, 175, 175], screen size 318 mm x 256 mm) with a size of 3° visual angle at 67.2 cm distance from the participant (Figure 1). During the ITI, a white (RGB: [255, 255, 255]) fixation cross (0.8° visual angle) was presented at the center of the same gray background screen.

An automatic pump (AL‐8000 Syringe Pump, World Precision Instruments, Sarasota FL, USA) dispensed US via a 5‐m PVC tube (Faust Laborbedarf AG, Schaffhausen, CH) with an inner diameter of 2 mm and an outer diameter of 4 mm. This tube was positioned in such a way that it sat easily within the participants' mouths and was affixed to a chin rest, on which participants were asked to place their chins throughout the task.

#### 2.2.4 Recording of Psychophysiological Indices

We collected ECG data with three pregelled Ag/AgCl adhesive snap electrodes (01–7500, TIGA‐MED; FS‐TC1, Skintact; and EL503, Biopac Systems Inc.) attached to the outsides of both wrists and the right ankle. For each participant, we recorded the lead configuration that yielded the clearest R spikes (ECG100C, Biopac Systems Inc.). To record skin conductance, two 8‐mm disk Ag/AgCl cup electrodes (EL258, Biopac, Goleta, CA) filled with 0.5% NaCl gel (GEL101, Biopac; Hygge & Hugdahl, 1985) were applied to the thenar and hypothenar eminence of the nondominant hand and connected to a constant voltage coupler/amplifier (EDA100C, Biopac). We measured respiration using a single‐belt cushion system (RSP100C, Biopac) attached around the chest at the lower end of the sternum. All data were amplified and digitized (MP160, Biopac) and recorded with Acknowledge (version 5.0, Biopac). Pupil size and gaze direction were recorded for both eyes with an Eyelink 1000 system (SR Research, Ottawa, ON, Canada) at a sampling rate of 500 Hz and an eyetracker‐participant distance of 52.2 cm, after gaze calibration using the manufacturer's 9‐point procedure.

### 2.3 Preprocessing and Modeling of Psychophysiological Indices

We analyzed all data in the framework of psychophysiological modeling (PsPM). Additionally, we report peak‐scoring analyses of pupil size responses and grand means of intratrial time courses.

#### 2.3.1 Psychophysiological Modeling

Psychophysiological (forward) models mathematically describe how a neural input generates a peripheral physiological response (Bach et al. 2018). The amplitudes of the input into this system are assumed to reflect the psychological latent variable, which in the context of associative learning is the CS–US association. Given a psychophysiological model, the latent variable can then be estimated from physiological data by means of model inversion (Bach et al. 2018). Similar to previous work on these observables (Bach, Flandin, et al. 2010; Castegnetti et al. 2016, 2017; Korn et al. 2017), we constructed our psychophysiological models as linear time‐invariant systems (LTI), which are fully defined by their response functions (RF). Two approaches are often used to construct the RF for an LTI. On the one hand, one may formalize the RF from identified biophysical relations between inputs and outputs. This approach assumes that researchers already understand the underlying physiology (Friston et al. 2000). On the other hand, one may construct a phenomenological RF from the empirical data, even if the underlying biophysical systems are unknown (Castegnetti et al. 2016). In the present work, we employed the second approach. For SCR, the existing phenomenological model is split into an invariant peripheral system that does not depend on the experimental paradigm, and a flexible model of the neural input, which can be estimated from data. The peripheral model has been validated by direct intraneural recordings (Gerster et al. 2018), such that there was no need to develop a new RF for SCR. On the other hand, the existing RF for HPR, PSR, and RAR collapse paradigm‐specific central processes and the peripheral system, such that they are not necessarily applicable to the current experimental paradigm. Thus, we used data from experiment 1 to develop RF for these modalities.

Once the shape of the RF is defined, the next step is to estimate the system's input to best explain data. To obtain input amplitude estimates, we inverted general linear convolution models (GLM) to fit the predicted timeseries (obtained through the convolution of the RF with a constant input shape) to the empirical data timeseries (Bach et al. 2018). The GLMs are either trial‐wise or condition‐wise, depending on the modality of the data. Trial‐wise GLMs were used for PSR, which have a time course that does not overlap between trials (Korn et al. 2017), whereas condition‐wise GLMs were used for HPR and RAR. For SCR, we conducted trial‐wise estimation using the nonlinear model in PsPM (Bach, Daunizeau et al. 2010). See section 2.3.3 below for details.

All preprocessing and modeling of psychophysiological data were conducted using the PsPM toolbox 4.1.1 (https://bachlab.github.io/PsPM/) (Bach et al. 2018) in MATLAB R2021a.

#### 2.3.2 Heart Period Responses (HPR)

Heart beats were detected in ECG signal by a modified version of the Pan‐Tompkins algorithm (Pan and Tompkins 1985) as implemented in PsPM (Paulus et al. 2016). The presence of artifacts was further controlled by visually inspecting each participant's timeseries, and removing artifacts due to clipping, movement, or electrode detachment. For each detected heart beat, we computed the preceding inter beat interval. Inter beat intervals corresponding to a heart rate outside 50–150 beats per minute were automatically excluded. In line with previous work (Paulus et al. 2016), our analyses were based on heart period rather than heart rate, because heart period and autonomic input are linearly related in stimulation studies (Berntson et al. 1995). The remaining data points were linearly interpolated in chronological time at 100 Hz and filtered with a 4th‐order bidirectional band‐pass Butterworth filter (cut‐off frequencies: 0.015–0.5 Hz) as in previous work (Castegnetti et al. 2016). To build the RF, we extracted trial‐wise segments, and baseline‐corrected single‐trial responses by subtracting the heart period average during 5 s before the CS onset (Pollatos et al. 2007). Afterwards, responses were averaged first within each condition, and then over participants. In line with previous work (Castegnetti et al. 2016), we fitted the difference between the mean over all CS+ and the mean over all CS− trials with a gamma probability density function (κ = 1.72, θ [s^−1^] = 0.14, c = 60.10, $t_{0}$ [s] = −17.61). As the duration of typical HPR is much longer than the CS–US interval, only CS+ trials not reinforced by a US were considered for modeling and analysis.

#### 2.3.3 Skin Conductance Responses (SCR)

SCR data quality was assessed by the SCR preprocessing function implemented in PsPM. Specifically, raw data outside 0.05–100 μS or with an absolute slope over 10 μS/s were automatically marked as missing data. The presence of artifacts was further controlled by visually inspecting each participant's SCR time series and removing artifacts due to clipping, movement, or electrode detachment. All such missing data were linearly interpolated for filtering and removed from analysis. Data were then filtered with a bidirectional 1st‐order band‐pass Butterworth filter with the cut‐off frequencies 0.0159 Hz and 5 Hz and downsampled to 10 Hz.

We then estimated conditioned and unconditioned responses using a nonlinear model implemented in PsPM (Bach 2014; Bach and Melinscak 2020). We modeled a response evoked by CS onsets with fixed latency, and another evoked by US or US omission. Amplitude estimates were normalized by dividing by the average of all CS− trials from the corresponding participant.

#### 2.3.4 Pupil Diameter

Pupil diameter data were converted to metric units and preprocessed with the algorithm implemented in PsPM (Kret and Sjak‐Shie 2019). This algorithm excludes data points outside the biological range of pupil size and its time derivative. Furthermore, it excludes isolated data points, outliers, and data points at the beginning and the end of temporal gaps, interpolates the data, and combines data from both pupils. Next, data points were excluded if the gaze point deviated more than 5° visual angle from the screen center (Korn et al. 2017). We then corrected for the pupil foreshortening error (Hayes and Petrov 2016) and downsampled to 100 Hz after a low‐pass filter with a cutoff of 50 Hz. We developed and tested several RF (Table 2). For all models, pupil diameter time series for each participant and each block were z‐scored. Then, the first 3 (RF 3, 4, and 5), 3.5 (RF 2 and 6) or 15 (RF 1) s of each trial were extracted, and each timepoint was averaged first within condition, then over all participants. In line with previous work (Korn et al. 2017), we fitted the difference between the mean over all CS+ and the mean over all CS− trials. For RF1 that extended beyond the CS–US interval, we only considered CS+ trials that were not reinforced by a US, to avoid contamination of the PSR by overlapping US responses. For RF2–6, we used all trials. We compared this approach to two standard peak‐scoring methods. For the first method (Finke et al. 2021), we extracted the preprocessed pupil size within each trial from CS onset to US onset, which spans a duration of 5 s. Next, we subtracted the baseline value from the pupil data. The baseline was defined as the mean pupil size, excluding any missing values, during the 1‐s period preceding each CS onset. From this baseline‐corrected pupil data, we took the maximum value as the peak‐scored pupil dilation of a trial. Trials with unavailable baseline (i.e., with all missing values during the 1‐s pre‐CS period) were marked as missing data (Finke et al. 2021). The second peak‐scoring method was similar with different time windows (Pietrock et al. 2019). Baseline correction spanned the 2‐s period preceding each CS onset, and the peak was defined as maximum during the 1‐s period before US onset (Pietrock et al. 2019).

**TABLE 2:** Pupil size response functions.

Model	Type	Specification
RF1	Gamma	κ = 3.534, θ [s^−1^] = 1.946, c = −1.183, $t_{0}$ [s] = 1.712
RF2	Gamma	κ = 30.781, θ [s^−1^] = 0.042, c = 0.033, $t_{0}$ [s] = 0.506
RF3	Gaussian	μ [s] = 1.784, σ = 0.246, c = 0.035
RF4	Low‐Pass Filtered	Bidirectional Butterworth 2 Hz Low‐Pass Filter of the first 2.88 s of the mean difference of CS+ and CS−
RF5	Mixture of two Gammas	c = 12.998, $o_{1}$ = 1.355, $o_{2}$ = 0.750, $d_{1}$ = 0.059, $d_{2}$ = 0.178, r = 2.715, $t_{0}$ = 0.439
RF6	Difference of the mean prediction of 20 Gaussian Processes fit to all CS+ and CS− trials separately	CS+: Matern kernel: ν = 1.5, l = 1.031 (*sd* = 0.000), white noise kernel: 0.922 (*sd* = 0.005) CS−: Matern kernel: ν = 1.5, l = 1.031 (*sd* = 0.000), white noise kernel: 0.903 (*sd* = 0.007)

*Note:* RF refers to response function.

#### 2.3.5 Respiration Amplitude (RAR)

To extract respiration amplitude from the raw time series, we used an established algorithm implemented in PsPM which automatically detects respiration cycles (Bach et al. 2016). The respiration amplitude values were interpolated at 10 Hz, and band‐pass filtered with a bidirectional Butterworth filter, with 2‐Hz low‐pass and 0.01‐Hz high‐pass cutoffs. As for the other modalities, and in line with previous work (Castegnetti et al. 2017), we fitted the difference between the mean over all CS+ and the mean over all CS− trials with a gamma pdf (κ = 40.87, θ[s^−1^] = 0.29, c = 0.14, $t_{0}$[s] = 2.09).

### 2.4 Statistical Analysis

All data analysis was conducted in MATLAB R2021a (The Math Works, https://www.mathworks.com/products/matlab.html) and R 4.2.1 (Ihaka and Gentleman 1996).

#### 2.4.1 Exclusion Criteria

For SCR, HPR, and RAR, we excluded participants if their estimated data were outside three standard deviations around the corresponding condition group mean. For PSR, trials with unreasonable pupil dilation estimates (estimates exceeding ±6 mm) (Spector 1990) or with more than 50% missing values were excluded, and participants were excluded if they had more than 50% of trials removed. Please see Table S1 for a summary of the number of excluded trials and participants for each measure in each phase for each experiment.

#### 2.4.2 Data Analysis

For all models in which the RF overlapped with the US presentation (HPR, HPR, RAR, PSR RF1), we retained data from CS− trials and nonreinforced CS+ trials only (to avoid biasing the estimated CRs by the US response). Next, we obtained condition‐wise estimates by averaging data across all trials within each CS condition. Finally, we performed pairwise *t*‐tests to examine the CS+/CS− difference.

As the recall test was done without US reinforcement, CS+/CS− differences are likely to extinguish over the course of the recall test. Thus, including all trials into the analysis might reduce a CS+/CS− difference seen in early trials. On the other hand, including fewer trials might increase the impact of trial‐by‐trial variation due to experiment‐unrelated factors, and the optimal balance is difficult to intuit (Khemka et al. 2017). Hence, for data available on a trial‐by‐trial basis, we approached this in a data‐driven way by analyzing the condition average over 1…*n* trials, with *n* ranging from 1 to the number of trials per condition. Similarly, at least for some observables, it is speculated that CS–US association is learned relatively quickly, but that the CS+/CS− differences might decay over time (Tzovara et al. 2018). Hence, we did a similar analysis for the learning phase, excluding the first pair of trials. For SCR and PSR RF1, where we retained only nonreinforced CS+ trials, we would average over 1…n CS+ trials and 2…2n CS− trials, where n ranges from 1 to the number of nonreinforced CS+ trials. For PSR (with the exception of RF1), CS+ refers to both reinforced and nonreinforced CS+ trials, for which the estimated CR does not overlap with US presentation. Finally, for observables unavailable on a trial‐by‐trial basis, we analyzed the first and the second half of the phases separately.

We computed effect sizes to compare and find the optimal psychophysiological measure(s) for reward learning. For all models, the Hedge's *g* was computed using the following formula (Lakens 2013).

$$g=\frac{t}{\sqrt{n}}∙\frac{\Gamma \left(\frac{n-1}{2}\right)}{\sqrt{\frac{n-1}{2}}\Gamma \left(\frac{n-1}{2}\right)}\;\left(1\right)$$

### 2.5 Data and Code Accessibility

Anonymised data is available on Zenodo (Experiment 1: https://doi.org/10.5281/zenodo.12580446; Experiment 2: https://doi.org/10.5281/zenodo.12580463). Experimental materials (stimuli and MATLAB scripts) and scripts of data analysis are available on OSF (https://osf.io/9s6uh/). An updated heart period response function, fitted on data from both experiments, is available in the PsPM toolbox from version 7 onwards as pspm_bf_hprf_rew (https://bachlab.github.io/pspm).

## 3 Results

### 3.1 Experiment 1

**FIGURE 2:**
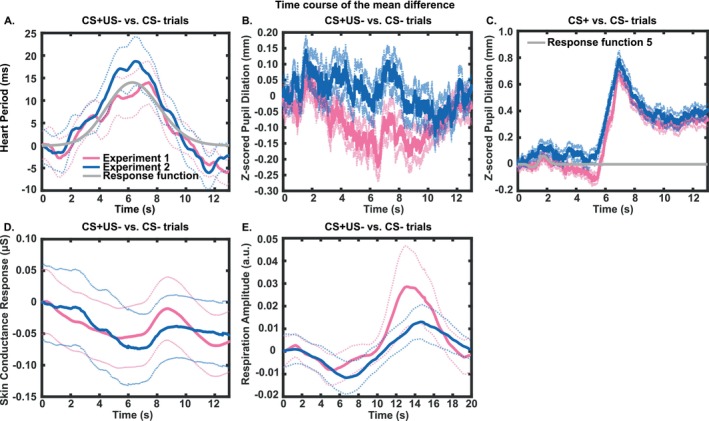
Responses in four modalities, and relevant response functions. All panels show the condition differences between CS+ and CS− trials during learning for experiments 1 (magenta) and 2 (blue). (A) HPR and corresponding RF (nonreinforced CS+ trials). (B) PSR (nonreinforced CS+ trials, used for RF1). (C) PSR (all CS+ trials, used for RF2‐6 and peak scoring) and RF5. (D) SCR (nonreinforced CS+ trials), and (E) RAR (nonreinforced CS+ trials). Solid lines indicate mean and dotted lines represent ± SEM. Data from experiment 2 are shown for illustration only and were not used to fit the response functions. *X*‐axis represents time since trial onset (i.e., CS onset); US onset is after 5 s.

**FIGURE 3:**
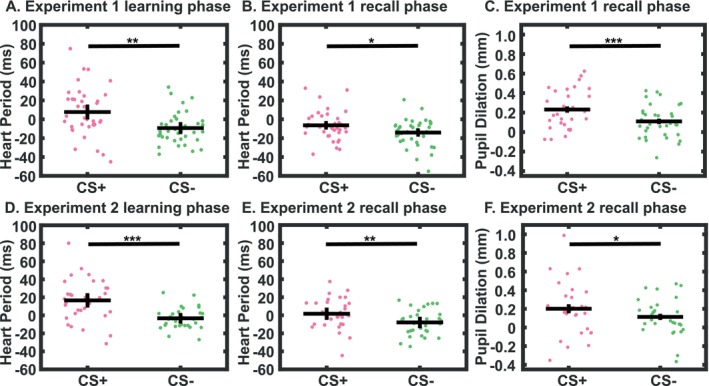
Condition‐wise mean heart period responses for learning phase (A) and recall phase (B) in experiment 1, as well as learning phase (D) and recall phase (E) in experiment 2; condition‐wise mean PSR (RF5) for the first 7 CS+ and 7 CS− trials of recall phase (C) in experiment 1, and recall phase (F) in experiment 2. CS+ refers to nonreinforced CS+ trials without US presence for HPR; and to all CS+ trials for PSR. The statistical test in A is biased (RF generated from the same data), while tests in B‐F are unbiased (RF generated from learning data in experiment 1). Dots represent individual condition‐wise estimates. Black crosses represent condition group mean ± SEM. *p < 0.05; **p < 0.01; ***p < 0.001 (uncorrected).

Grand means for the intratrial time course, as well as the two relevant RF, are shown in Figure 2. In the learning phase, the condition difference between CS+ and CS− exceeded our a priori effect‐size threshold (Hedge's *g* > 0.5) for the HPR index only (Table 3, Figure 3). For PSR, indices from RF4 and RF5 were close to the threshold with *g*‐values around 0.4, while there was no apparent CS+/CS− difference for SCR and RAR (Table 3). Notably, this analysis is biased for HPR, PSR, and RAR because it is based on RF fitted to the same data sets. Finally, there was no CS+/CS− difference in either peak‐scoring analysis of PSR. Analysis of trial‐by‐trial responses revealed no subsets of trials that exceeded the threshold in any PSR metric or SCR, and there were no additional insights from separately analyzing the first and second halves of the learning phase for HPR and SCR.

**TABLE 3:** Results of pairwise *t*‐tests for HPR, SCR, PSR, and RAR.

Phase	Condition, comparison	HPR	SCR	PSR RF1	PSR RF2	PSR RF3	PSR RF4	PSR RF5	PSR RF6	PSR peak scoring 1	PSR peak scoring 2	RAR
Learning	CS+	7.69 (26.17)	0.96 (0.15)	0.01 (0.08)	0.05 (0.16)	0.05 (0.16)	0.10 (0.17)	0.10 (0.15)	−0.006 (0.16)	0.30 (0.10)	0.11 (0.09)	−0.01 (0.03)
(Exp1)	CS−	−9.34 (16.73)	0.95 (0.11)	−0.02 (0.07)	0.02 (0.14)	0.03 (0.14)	0.05 (0.15)	0.06 (0.14)	−0.02 (0.14)	0.30 (0.09)	0.12 (0.08)	−0.01 (0.03)
	CS+ vs. CS−	*t* (36) = 3.51, *p* = 0.001**, *g* = 0.56	*t* (36) = 0.42, *p* = 0.68, *g* = 0.07	*t* (33) = 1.62, *p* = 0.12, *g* = 0.27	*t* (33) = 1.53, *p* = 0.14, *g* = 0.26	*t* (33) = 1.19, *p* = 0.24, *g* = 0.20	*t* (33) = 2.24, *p* = 0.03, *g* = 0.39	*t* (33) = 2.15, *p* = 0.04, *g* = 0.36	*t* (33) = 0.74, *p* = 0.46, *g* = 0.12	*t* (36) = −0.33, *p* = 0.74, *g* = −0.05	*t* (36) = −1.10, *p* = 0.28, *g* = −0.18	*t* (34) = 0.39, *p* = 0.70, *g* = 0.06
Recall (Exp1)	CS+	−6.42 (15.47)	0.93 (0.21)	−0.03 (0.07)	0.04 (0.14)	0.04 (0.15)	0.11 (0.16)	0.09 (0.13)	−0.001 (0.15)	0.32 (0.08)	0.17 (0.07)	
	CS−	−14.07 (14.51)	0.92 (0.18)	−0.03 (0.08)	0.03 (0.10)	0.03 (0.10)	0.09 (0.15)	0.07 (0.12)	0.004 (0.12)	0.31 (0.08)	0.15 (0.08)	
	CS+ vs. CS−	*t* (36) = 2.48, *p* = 0.02*, *g* = 0.40	*t* (36) = 0.50, *p* = 0.62, *g* = 0.08	*t* (34) = −0.38, *p* = 0.71, *g* = −0.06	*t* (34) = 0.19, *p* = 0.85, *g* = 0.03	*t* (34) = 0.24, *p* = 0.81, *g* = 0.04	*t* (34) = 0.91, *p* = 0.37, *g* = 0.15	*t* (34) = 0.97, *p* = 0.34, *g* = 0.16	*t* (34) = −0.24, *p* = 0.81, *g* = −0.04	*t* (36) = 0.66, *p* = 0.51, *g* = 0.11	*t* (36) = 1.32, *p* = 0.19, *g* = 0.21	
Learning (Exp2)	CS+	16.57 (22.90)	0.97 (0.11)	0.02 (0.11)	0.03 (0.14)	0.03 (0.14)	0.06 (0.19)	0.07 (0.15)	−0.002 (0.16)	0.28 (0.10)	0.11 (0.08)	0.01 (0.04)
	CS−	−3.29 (11.26)	0.99 (0.03)	0.01 (0.07)	0.01 (0.10)	0.01 (0.10)	0.04 (0.13)	0.05 (0.11)	−0.02 (0.12)	0.28 (0.09)	0.09 (0.08)	−0.01 (0.03)
	CS+ vs. CS−	*t* (32) = 4.56, *p* < 0.001***, *g* = 0.78	*t* (33) = −0.84, *p* = 0.41, *g* = −0.14	*t* (31) = 0.41, *p* = 0.68, *g* = 0.07	*t* (30) = 0.83, *p* = 0.41, *g* = 0.15	*t* (30) = 1.23, *p* = 0.23, *g* = 0.22	*t* (31) = 0.76, *p* = 0.45, *g* = 0.13	*t* (30) = 0.83, *p* = 0.41, *g* = 0.15	*t* (31) = 0.64, *p* = 0.53, *g* = 0.11	*t* (33) = 0.54, *p* = 0.59, *g* = 0.09	*t* (33) = 1.11, *p* = 0.27, *g* = 0.19	*t* (32) = 1.98, *p* = 0.06, *g* = 0.34
Recall (Exp2)	CS+	1.66 (16.31)	0.93 (0.14)	−0.02 (0.06)	0.02 (0.12)	0.02 (0.12)	0.04 (0.14)	0.04 (0.12)	−0.01 (0.15)	0.28 (0.09)	0.12 (0.09)	
	CS−	−7.96 (13.55)	0.97 (0.08)	−0.03 (0.08)	0.02 (0.11)	0.02 (0.11)	0.04 (0.14)	0.05 (0.11)	−0.02 (0.13)	0.28 (0.08)	0.11 (0.08)	
	CS+ vs. CS−	*t* (32) = 3.26, *p* = 0.002**, *g* = 0.55	*t* (32) = −1.67, *p* = 0.10, *g* = −0.28	*t* (31) = 0.58, *p* = 0.57, *g* = 0.10	*t* (31) = 0.08, *p* = 0.93, *g* = 0.01	*t* (31) = 0.25, *p* = 0.81, *g* = 0.04	*t* (31) = 0.13, *p* = 0.90, *g* = 0.02	*t* (31) = −0.19, *p* = 0.85, *g* = −0.03	*t* (31) = 0.66, *p* = 0.51, *g* = 0.11	*t* (32) = 0.30, *p* = 0.77, *g* = 0.05	*t* (32) = 0.11, *p* = 0.91, *g* = 0.02	

*Note:* RF refers to response function. Rows labeled CS+ and CS− show group mean (standard deviation). Rows labeled CS+ vs. CS− shows degrees of freedom in parentheses. CS+ refers to both reinforced and nonreinforced CS+ trials in columns PSR RF2‐6 and PSR peak scoring, and to nonreinforced CS+ trials otherwise. *p*‐values are uncorrected and presented for illustration only; our a priori decision criterion to retain an index was based on Hedge's *g*.

*p < 0.05; **p < 0.01; ***p < 0.001.

For the recall phase, we found an above‐threshold condition difference between CS+ and CS− for HPR (Table 3, Figure 3). Because the RF was fit on data from the learning phase only, this was an unbiased analysis and therefore suggests that participants indeed retained reward memory. When averaging over all trials, there were no CS+/CS− differences in any other metric. However, trial‐by‐trial analysis of PSR revealed large effects in the first part of the recall phase. The largest effect size was achieved by PSR RF5 for the first 7 CS+ and CS− trials (*g* = 0.68) (Figure 3). In SCR, effect sizes up to around *g* = 0.35 were observed in the first 3–4 CS+ and CS− trials. See Appendix S1 for trial‐by‐trial results for all metrics from the recall phase.

### 3.2 Experiment 2

We analyzed experiment 2 with RF developed in experiment 1, so this represents an unbiased out‐of‐sample generalization analysis. For HPR, the condition differences between CS+ and CS− were significant for learning and recall after Holm‐Bonferroni correction for multiple comparisons across two tests (learning and retention); see Table 3 and Figure 3 for details. For PSR, the effects observed in the learning phase of experiment 1 in RF4/5 were not replicated; effect sizes were *g* < 0.15 when averaging over all trials. The trial‐by‐trial analysis that yielded the largest effect size for recall in experiment 1 (PSR RF5 for the first 7 CS+ and CS− trials) also yielded the largest effect size in experiment 2 (*g* = 0.41, *p* < 0.05, Figure 3, see also Appendix S1 for details). Significant trial‐by‐trial results observed for SCR in experiment 1 were not replicated in experiment 2.

### 3.3 Subjective Ratings

Table 4 displays the summary statistics for post‐experiment questionnaire data in experiments 1 and 2. Overall, the data patterns are consistent across these two experiments. The contingency awareness and valence were higher for CS+ (vs. CS−) in both learning and recall phases; the arousal was larger for CS+ (vs. CS−) in the learning phase only.

**TABLE 4:** Summary statistics and results of paired *t*‐tests for questionnaire data in experiments 1 and 2.

Experiment	Phase	Condition, comparison	Contingency awareness (%)	Arousal rating (%)	Valence rating (%)
1	Learning	CS+	45.92 (32.66)	54.36 (29.41)	58.59 (22.38)
		CS−	10.85 (21.99)	26.12 (23.55)	38.80 (19.18)
		CS+ vs. CS−	*t* (36) = 17.50, *p* < 0.001***	*t* (36) = 10.36, *p* < 0.001***	*t* (36) = 3.28, *p* < 0.01**
1	Recall	CS+	48.18 (33.47)	2.99 (7.62)	35.28 (26.12)
		CS−	13.73 (19.44)	1.33 (4.53)	12.60 (14.75)
		CS+ vs. CS−	*t* (36) = 9.86, *p* < 0.001***	*t* (36) = 2.01, *p* = 0.05	*t* (36) = 4.59, *p* < 0.001**
2	Learning	CS+	42.73 (33.70)	50.88 (29.83)	59.69 (21.26)
		CS−	6.37 (11.42)	21.39 (14.35)	32.85 (18.05)
		CS+ vs. CS−	*t* (33) = 16.48, *p* < 0.001***	*t* (33) = 11.08, *p* < 0.001***	*t* (33) = 4.69, *p* < 0.001***
2	Recall	CS+	44.71 (33.61)	1.12 (3.13)	31.35 (27.89)
		CS−	9.41 (24.08)	4.24 (12.87)	25.17 (21.84)
		CS+ vs. CS−	*t* (32) = 12.55, *p* < 0.001***	*t* (32) = −0.82, *p* = 0.42	*t* (32) = 6.30, *p* < 0.001***

*Note:* The numbers in the columns contingency awareness, arousal rating, and valence rating are condition means and standard deviations inside the parentheses.

**p < 0.01; ***p < 0.001 (uncorrected).

## 4 Discussion

Pavlovian reward conditioning is an important basic learning paradigm, but its optimal quantification in humans remains unclear, in particular for recall after overnight consolidation. Here, we pitched different CRs against each other in the same experimental paradigm. We used a rigorous exploration‐confirmation approach to establish the best psychophysiological indices for measuring reward learning and memory retention over 7 days. Our key finding is that among candidate psychophysiological indices (based on HPR, SCR, PSR, and RAR), model‐based HPR analysis distinguished CS+/CS− in the learning phase across both experiments, with Hedge's *g* = 0.56 in experiment 1 and *g* = 0.78 in experiment 2. It also distinguished CS+/CS− in the recall test after 7 days, with *g* = 0.40 in experiment 1 and *g* = 0.55 in experiment 2. Furthermore, model‐based PSR analysis distinguished CS+/CS− in the recall phase with the largest effect size when averaging over 7 trials per condition in experiment 1 (*g* = 0.69) and experiment 2 (*g* = 0.41). Thus, HPR and PSR as analyzed with our new response functions appear to be replicable and valid measures of classical reward conditioning and memory retention over 7 days.

### 4.1 HPR Discriminated CS+/CS− Difference in Learning and Recall

HPR has been examined in previous reward conditioning work; however, with conflicting results. Our present observation of reward‐conditioned bradycardia replicates a previous study (Pietrock et al. 2019). This work employed a highly similar Pavlovian reward conditioning paradigm and analyzed HPR using PsPM's general linear convolution model (GLM). In contrast, in other previous work, cardiac responses did not discriminate CS+/CS− (Ebrahimi et al. 2019; Exner et al. 2021; Hermann et al. 2000; Sayão et al. 2021; Wardle et al. 2018). There appear to be two main differences between these studies and ours. First, these studies used heart rate as the CR and/or analyzed HPR using different approaches (e.g., mean change, heart index, and mean level) (Ebrahimi et al. 2019; Sayão et al. 2021; Wardle et al. 2018). Compared to heart rate, HPR has been shown to linearly relate to neural input into the heart and is therefore more likely to linearly relate to psychological variables (Berntson et al. 1995). Also, work on fear conditioning suggests that a model‐based approach might be more sensitive to discriminate CS+/CS− differences based on HPR than peak‐scoring analysis (Castegnetti et al. 2016; Paulus et al. 2016). Hence, the observed null results in previous studies may be due to the selection of the CR index. Second, in some studies using odor as the US, a conditioning effect was not only absent for the HPR but, in fact, for all psychophysiological measures (Exner et al. 2021; Hermann et al. 2000). A potential interpretation is that participants simply did not learn, possibly due to reduced associability of CS and odor (Kokkola et al. 2019).

**FIGURE 4:**
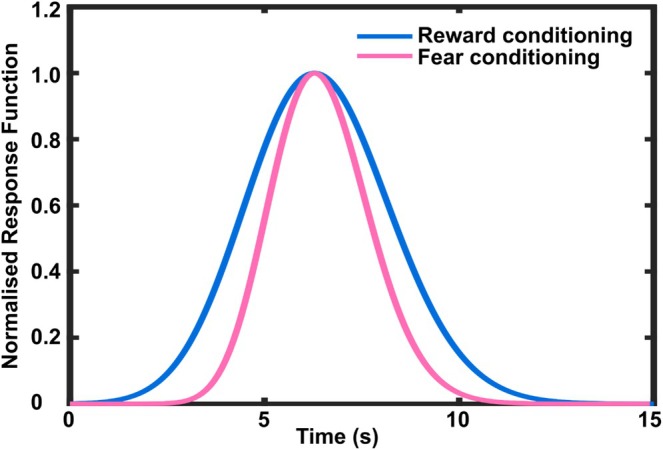
Heart period response function (normalized) with the same SOA (5 s) for reward and fear conditioning, respectively.

Another interesting question is whether reward and fear conditioning affect HPR differently. Descriptively, the effect size for reward‐conditioned HPR (Cohen's *d* = 0.79 in learning phase of experiment 2) is smaller than that of fear‐conditioned HPR (Cohen's *d* = 0.97) (Bach and Melinscak 2020). Also, the response functions differ from one another (RF for reward conditioning: κ = 1.72, θ [s^−1^] = 0.14, c = 60.10, $t_{0}$ [s] = −17.61; RF for fear conditioning: κ = 48.5, θ [s^−1^] = 0.182, c = 1, $t_{0}$ [s] = −7.36) (Figure 4). Compared to reward conditioning, the fear‐conditioned response appears narrower and returns to baseline more quickly. This could suggest a more vigilant preparatory reaction to successive behavioral response, which might be evolutionarily adaptive (Andreatta and Pauli 2015). Finally, there is evidence that fear‐conditioned bradycardia is time‐locked to US onset, that is, the onset of fear‐conditioned bradycardia moves as the time point of possible US delivery changes after CS onset (Castegnetti et al. 2016, 2017). In the present work, CS–US interval was not varied, such that we could not investigate this question for reward conditioning. Overall, it remains unclear whether reward‐ and fear‐conditioned HPR operate through different mechanisms. Fear‐conditioned bradycardia has been linked to behavioral freezing (immobility), which might have adaptive value in certain defensive situations (Roelofs and Dayan 2022), although we note other work has demonstrated freezing in conjunction with tachycardia as well as with bradycardia (Signoret‐Genest et al. 2023), such that this link remains speculative.

Our recall test after 7 days revealed retention of the conditioned HPR in both experiments. To our best knowledge, no prior studies have assessed the retention of human reward learning memory after a delay of several days. The recall phase can be viewed as an extinction training due to the lack of reinforcers. Hence, it is interesting that there appears to be only limited extinction for reward learning. Several reasons might be plausible. First, previous research on fear learning found that HPR is more resistant to extinction compared to SCR and saccadic scanpath length (Xia et al. 2021). It is possible that in the current paradigm, the number of trials (96) is not sufficient to extinguish reward conditioning. Another potential explanation is that HPR becomes habitual during learning and looses its dependence on US outcome predictions (Pool et al. 2019).

### 4.2 SCR Did Not Discriminate CS+/CS− Difference in Learning

Interestingly, we did not find evidence that SCR discriminated CS+/CS− difference during the learning phase, although this measure was indeed sensitive to reward conditioning in most previous work (Andreatta and Pauli 2015, 2019; Ebrahimi et al. 2019; Klucken et al. 2019; Kruse et al. 2017, 2020; Tapia León et al. 2018; van den Akker et al. 2017; Wardle et al. 2018). Upon a closer look, it seems that the US type and response quantification approach influenced whether SCR discerned CS+/CS− difference. There were two main US types (primary reinforcers such as snacks, fruit juice; secondary reinforcers such as monetary reward) and two response quantification approaches (model‐based approach such as psychophysiological models, nonmodel‐based approach such as through‐to‐peak approach) in previous work (Andreatta and Pauli 2019; Ebrahimi et al. 2019; Kruse et al. 2020; van den Akker et al. 2017). All previous work employing nonmodel‐based approaches, particularly the through‐to‐peak and baseline‐to‐peak methods, has found CS+/CS− difference in SCR, regardless of the US type (Andreatta and Pauli 2015, 2019; Klucken et al. 2019; Kruse et al. 2017, 2020; Tapia León et al. 2018; Wardle et al. 2018). In contrast, research utilizing model‐based approaches (PsPM GLM and Ledalab), with primary and secondary reinforcers, presents mixed evidence. Among these, only one study out of five (including the present work) has found a difference in SCR between CS+ and CS− (Ebrahimi et al. 2017, 2019; Pietrock et al. 2019; van den Akker et al. 2017). Collectively, these findings could suggest that model‐based approaches may be less effective in detecting CS+/CS− differences in SCR, for example, because they did not assume the correct underlying model of SCR generation. However, the heterogeneity of peak‐scoring schemes in previous work and the limited number of studies warrant caution. Another interpretation is that different primary reinforcers (fruit juice in the present work, snacks in some previous work) elicit different CRs, potentially due to differences in arousal elicited by the US.

### 4.3 PSR Discriminated CS+/CS− Difference in Recall but Not During Learning

Unexpectedly, we did not find replicable CS+/CS− differences based on PSR during learning (Bray et al. 2008; Pietrock et al. 2019; Pool et al. 2019; Prévost et al. 2013; Seymour et al. 2007), despite our reward‐conditioning paradigm being closely modeled on Pietrock et al. (2019), who did report a difference. There are two main differences between our study and theirs. One is the trial‐by‐trial collection of US expectancy ratings in their study. This might strengthen contingency awareness, which in turn could affect PSR (Van Dessel et al. 2019). The second is the CS sensory modality. Pietrock et al. (2019) used compound CS (i.e., visual and auditory stimuli presented simultaneously). Finally, other previous reward‐conditioning studies that revealed CS+/CS− differences for PSR employed a reinforcement rate larger than the 50% used here (O'Doherty et al. 2003; Pool et al. 2019; Prévost et al. 2013; Reinhard and Lachnit 2002; Schad et al. 2020). On the other hand, PSR distinguished CS+/CS− early during recall, with the largest effect size in both experiments when averaging over 7 CS+ and 7 CS− trials. How this discrepancy between initial learning and recall can be reconciled is unclear at this point.

### 4.4 Future Directions

The present work raises several important questions. First, it remains unclear how reward and fear conditioned HPR differ from each other, and what their adaptive value might be. Second, the variability of paradigm characteristics may influence the reliability of measurement. Future work may systematically examine the roles of these characteristics (e.g., reinforcement rate, US expectancy, type of CS and US stimuli, SOA, etc.) in reward learning. It might be useful to capitalize on a consensus paradigm in an experiment‐based calibration approach, as has been proposed for aversive conditioning (Bach et al. 2023). Third, in both of our experiments, the effect size of the HPR largely decreased from the learning to the memory retention phase. On the other hand, PSR robustly differentiated CS+/CS− during recall but not early learning. Hence, future research could investigate the temporal dynamics of PSR and HPR as markers of reward learning.

## 5 Conclusion

In conclusion, we identified HPR as a robust marker of reward learning and retention of reward memory, as well as PSR as a robust marker of reward memory. These findings may be beneficial for studies that involve reward learning and memory processes. Our work may also facilitate the identification of individuals exhibiting atypical reward learning patterns, thereby enabling the development of targeted treatment strategies.

## Author Contributions

**Yanfang Xia:** conceptualization, data curation, formal analysis, methodology, project administration, software, visualization, writing – review and editing. **Huaiyu Liu:** formal analysis, writing – original draft, writing – review and editing. **Oliver K. Kälin:** formal analysis, investigation, methodology, software. **Samuel Gerster:** methodology, software. **Dominik R. Bach:** conceptualization, funding acquisition, methodology, project administration, supervision, writing – review and editing.

## Conflicts of Interest

The authors declare no conflicts of interest.

## Supporting information


Appendix S1.

## Data Availability

Anonymised data is available on Zenodo (Experiment 1: https://doi.org/10.5281/zenodo.12580446 Experiment 2: https://doi.org/10.5281/zenodo.12580463). Experimental materials (stimuli and MATLAB scripts), and scripts of data‐analysis are available on OSF (https://osf.io/9s6uh/). An updated heart period response function, fitted on data from both experiments, is available in the PsPM toolbox from version 7 onwards as pspm_bf_hprf_rew (https://bachlab.github.io/pspm).
